# Neural Processing of Disorder-Related Stimuli in Patients with Anorexia Nervosa: A Narrative Review of Brain Imaging Studies

**DOI:** 10.3390/jcm8071047

**Published:** 2019-07-18

**Authors:** Joe J. Simon, Marion A. Stopyra, Hans-Christoph Friederich

**Affiliations:** Department of General Internal Medicine and Psychosomatics, Centre for Psychosocial Medicine, University Hospital Heidelberg, 69120 Heidelberg, Germany

**Keywords:** anorexia nervosa, functional magnetic resonance imaging, disorder-specific stimuli, narrative review

## Abstract

Abnormalities and alterations in brain function are commonly associated with the etiology and maintenance of anorexia nervosa (AN). Different symptom categories of AN have been correlated with distinct neurobiological patterns in previous studies. The aim of this literature review is to provide a narrative overview of the investigations into neural correlates of disorder-specific stimuli in patients with AN. Although findings vary across studies, a summary of neuroimaging results according to stimulus category allows us to account for methodological differences in experimental paradigms. Based on the available evidence, the following conclusions can be made: (a) the neural processing of visual food cues is characterized by increased top-down control, which enables restrictive eating, (b) increased emotional and reward processing during gustatory stimulation triggers disorder-specific thought patterns, (c) hunger ceases to motivate food foraging but instead reinforces disorder-related behaviors, (d) body image processing is related to increased emotional and hedonic reactions, (e) emotional stimuli provoke increased saliency associated with decreased top-down control and (f) neural hypersensitivity during interoceptive processing reinforces avoidance behavior. Taken together, studies that investigated symptom-specific neural processing have contributed to a better understanding of the underlying mechanisms of AN.

## 1. Introduction

Anorexia nervosa (AN) is a serious mental disorder characterized by self-induced starvation and excessive weight loss, fear of weight gain, body image concerns and food aversion [1]. Psychiatric comorbidities are common in patients with AN, as well as an increased mortality rate due to medical complications and suicide [2,3]. Previous research has identified numerous factors involved in the etiology of AN, where psychological, sociocultural and biological factors contribute to both the onset and maintenance of this disorder [4,5]. Recently, neurobiological alterations have been proposed as major factors contributing to AN [6]. Specifically, various studies have begun to employ neuroimaging techniques to elucidate the underlying pathophysiology and neurobiological substrate of AN [7,8,9,10]. Altered neural activity is observed throughout the brain in patients with AN, including cortical- and subcortical regions [7,11,12]. Based on neuroimaging investigations, theories have been proposed to explain the contribution of aberrant brain function to the development and maintenance of AN. For example, hyperactivity in cognitive control networks and a cooccurring reduction in motivational responses to food has been proposed as a core neural mechanism underlying the development of AN [5,12,13,14]. In contrast, reduced somatosensory and insula processing of taste stimuli may relate to a failure to accurately recognize hunger signals [15,16]. However, due to methodological differences between neuroimaging studies, as well as a paucity of studies employing a longitudinal design to differentiate between the state and trait, the exact neurobiological mechanisms of AN remain unclear. The aim of this review is to provide a narrative overview of recent studies investigating alterations in brain function related to disorder-specific stimuli in patients with AN and to provide a better characterization of the pathophysiological mechanisms underlying AN. Specifically, we focused on neuroimaging studies employing experimental designs drawing on symptom provocation to assess neural aberrations associated with AN. Symptom provocation has been extensively analyzed in patients with AN since the advent of fMRI-techniques [17] and has played an important role in the elaboration of neurobiological theories of AN. The following section will outline results from previous neuroimaging investigations grouped by stimulus type and relate these findings to current approaches examining neuroanatomical biomarkers of AN. Specifically, we describe studies investigating the following: (1) the responsivity to food-related stimuli, (2) hunger, (3) body image, (4) emotional processing and (5) interoceptive processing.

## 2. Neural Processing of Food-Related Stimuli

Food restriction and avoidance are cornerstones of AN, since patients are able to limit food intake even in the presence of prolonged food deprivation. Although the causative mechanisms are not completely understood, a relation to increased inhibition, alterations in the rewarding effect of food and a conditioned relationship between food and aversive emotional states have all been proposed as possible explanations [13,18]. Numerous studies have investigated brain alterations related to the exposure to different types of food stimuli in patients with AN. The experimental paradigms employed in these studies have been broadly classified as using either visual depictions of food or gustatory stimulation. 

### 2.1. Visual Food Stimuli

The majority of studies investigating the neural processing of food have employed visual depictions of food [19,20]. Visual stimuli are used in a wide array of different experimental tasks, and they allow researchers to probe psychopathology-related neural processing in an efficient and economical manner.

Previous studies employing *passive viewing of visual food stimuli* have yielded conflicting results, since a number different processes may be captured when passively viewing food stimuli [21]. However, altered activation of the amygdala and insula has been consistently observed in patients with AN during passive viewing of visual food stimuli [17,22,23]. Holsen and colleagues [24] investigated neural processing when patients viewed high- and low calorie food images and the relation to hormone mediated hunger signaling (i.e., by assessing the level of the hunger-inducing peptide hormone Ghrelin). They aimed to investigate potential neurobiological mechanisms underlying appetite dysregulation in AN. The authors observed a strong connection between hormone-mediated hunger signaling and the neural processing of food pictures in the amygdala and insula in healthy controls but were unable to detect the same relation in patients with acute AN and weight-recovered AN. These results suggest a link between the often observed resistance to hunger-inducing hormones in patients with AN and reduced motivational processing of food as a mechanism of restrictive eating.

When instructing participants to *imagine eating the depicted food stimuli* or to *rate stimuli* according to their pleasantness, patients with AN commonly display aberrant activation in different regions of the prefrontal cortex [25,26,27,28,29]. Specifically, as outlined in two recent reviews [20,21], hypoactivity during food picture processing in regions such as the inferior parietal lobule and lateral prefrontal cortex may indicate weight and body shape concerns induced by the exposure to visual food stimuli [27,30,31]. The cooccurring increased activation of medial prefrontal regions during picture processing may be related to increased top-down control and efforts to restrain eating and food avoidance [27,30,32,33]. For example, Scaife and colleagues [28] instructed patients with AN to look at images of food with a high caloric content and to focus on how much they want to eat the depicted items. As predicted, patients displayed increased activation in inhibitory brain regions (i.e., the lateral prefrontal cortex), consistent with the persistent avoidance of high-calorie food observed in patients with AN. These results are in line with eye-tracking data showing avoidance of food pictures by patients with AN [34]. Finally, decreased activation in the medial orbitofrontal cortex and insula during food picture processing has been interpreted as reduced hedonic reactivity to food stimuli in in patients with AN [20,31]. 

Foerde and colleagues [35] asked patients with AN to *choose between different visually depicted food items to assess the neural correlates of disorder-specific food choices in these patients*. When choosing low-fat foods, patients with AN displayed an increased connectivity between the dorsal striatum and dorsolateral prefrontal cortex [35]. Furthermore, this association was related to subsequent food intake, where a stronger connectivity was coupled with lower caloric intake on the following day [35]. The authors concluded that frontal-striatal networks are crucial in the development of habitual behaviors [36] and may subserve maladaptive eating behavior in patients with AN [35]. However, the authors did not clearly determine whether this observation was a risk factor for the development of AN or is simply caused by prolonged periods of self-starvation, particularly since adolescent patients with AN display hyperactivation in both reward-related and inhibitory control regions when viewing pictures of high calorie food [37].

Taken together, the results from studies investigating visual food stimuli note a pronounced top-down control mediated by medial prefrontal regions, which may override both somatosensory and hedonic-related brain signals.

### 2.2. Gustatory Stimulation

Studies employing real taste stimuli are able to probe disorder-specific neural reactions to food in a more natural setting. Behavioral investigations using gustatory stimulation have reported lower taste sensitivity in patients with AN [38], although patients report an increased subjective perception of taste stimuli [39]. Similarly, reduced taste classification accuracy in the insula during the tasting of sucrose has been observed in patients with AN [40], although conflicting results exist [32].

The majority of studies using gustatory stimulation to investigate the neural processing of taste found altered activation profiles in patients with AN compared to healthy controls. Interestingly, with a few exceptions, (e.g., [41]), most studies consistently detected increased activation in reward-related regions in patients with AN [15,42]. For example, Cowdrey and colleagues [32] compared the neural responses to rewarding and aversive tastes in participants who had recovered from AN and healthy controls. They did not observe differences between groups in subjective ratings of pleasantness and taste processing in the primary gustatory cortex, which indicates similar sensory experiences in both groups. However, the authors observed increased activation of brain regions processing reward and aversion (i.e., the ventral striatum and posterior insula, respectively) in response to both pleasant and aversive tastes. 

Increased activation of reward-related brain regions during taste processing has been discussed as an increased salience attribution to taste stimuli and is considered a potential neural biomarker for AN [32]. Similarly, Frank and colleagues observed increased reward-related activity during the processing of taste in both adolescent and adult patients with AN [43,44]. They propose a conflict between an innate starvation-induced approach mechanism to food and a strong drive for thinness. Specifically, the authors suggest that a starvation-induced sensitization of the dopamine system stimulates food intake, which is in direct conflict with a high drive for thinness and body dissatisfaction. Thus, neural reward processing may then become associated with a fear-driven mechanism that overrides homeostatic signals that would normally initiate feeding behavior [43]. These results are in part corroborated by a study by Vocks and colleagues [15], where patients with AN displayed higher activation in the amygdala and medial temporal gyrus when drinking chocolate milk than healthy controls during hunger. Since the amygdala is related to the processing of aversive stimuli and in the acquisition of conditioned emotional responses [45], the authors postulated that this finding indicated an increased fear of weight gain.

Horndasch et al. [46] used an incentive delay task, allowing the measurement of both anticipation and receipt of pleasant and aversive tastes. In a group of first-degree relatives of patients with AN, the authors observed increased neural reward processing during the anticipation of both pleasant and unpleasant tastes, but they observed a decreased reward processing during the receipt of aversive taste. They interpret this finding as increased emotional arousal in response to food anticipation and a bias towards reduced liking of food, with both observations representing a potential biomarker for AN. 

Taken together, the results from studies investigating taste processing indicate an increased salience of taste stimuli in patients with AN, which appears to be strongly correlated with disorder-specific reactions to food stimuli, such as fear of weight gain and a drive for thinness.

## 3. Hunger

As stated above, since self-starvation allows the maintenance of the desired low body weight, this behavior develops rewarding qualities in patients with AN and reinforces the illness [13]. Hunger promotes foraging by inducing increased mesolimbic dopaminergic signaling, which increases the motivational value of foraging behaviors [47]. This mechanism is thought to facilitate the progression to anorexia nervosa, where the constant fasting-induced dopamine stimulation reinforces disorder-related behaviors that are otherwise perceived as aversive [48,49,50]. However, few neuroimaging studies have directly compared the effects of both hunger and satiety on the processing of disorder-related stimuli in patients with AN. 

Using *visual depictions of food*, Santel and colleagues [51] observed decreased somatosensory processing during satiation in the parietal cortex and decreased processing in the visual cortex during hunger when subjected rated food pictures. Similarly, when passively viewing food pictures, Gizewski and colleagues [52] observed satiety state-dependent differences in brain activation in the cingulate cortex, insula and prefrontal cortex in patients with AN compared to healthy controls. Furthermore, both patients with AN and weight-restored patients show a general hypo-activation pattern in regions associated with motivational processing when viewing pictures of food while they are hungry [53]. The authors conclude that hunger loses its ability to induce an increased motivational drive for food consumption, thereby facilitating food restriction. 

By presenting *taste stimuli* during both hunger and satiety, Vocks and colleagues [15] found that patients with AN display increased activation in the amygdala and medial temporal gyrus while tasting chocolate milk during hunger. Within the patient group, satiety led to an increased activation of the right inferior temporal gyrus, a region that is typically activated during the processing of body images, compared to the hungry state [54]. Together with the observed activation of the amygdala and its relevance to the processing of aversive and fear-inducing stimuli [45,55], the authors propose that the observed results possibly reflect a fear of weight gain in patients with AN.

Taken together, studies investigating the effect of hunger on brain activation in patients with AN observe decreased activation in areas related to motivational processing and an interaction with emotional processing related to a fear of weight gain. However, these results relate exclusively to visual and gustatory food cues, since the influence of the satiety state on the processing of additional disorder-related stimuli remains to be investigated.

## 4. Body Image 

Body image distortion is a hallmark feature of AN. The subjective perception of body weight or shape is disturbed, together with increased attention to particular details or parts of the body [1]. Body image disturbances are persistent symptoms of AN and are negatively correlated with patients’ long-term outcomes [56]. In healthy participants, a network of brain regions is associated with body image processing. Studies investigating brain activation when participants compare their own body with slim-idealized bodies or when they are presented with distorted images of their own body typically observe increased activation in regions such as the extrastriate body area (a subportion of the extrastriate visual cortex), the fusiform body area located in the fusiform gyrus [54,57], the dorsolateral prefrontal cortex and parietal lobe [58]. Previous studies have observed alterations in this network in patients with AN, although the direction of differences has been inconsistent [59,60,61,62]. For example, when viewing body images of other women, patients with AN display both similar and differing brain activation patterns compared with healthy controls [59,63]. Furthermore, studies have observed both activation in fear-related networks [64,65], and reward-related networks during body image processing [66]. 

During the *passive viewing of body images*, patients with AN display reduced connectivity between the extrastriate and fusiform body areas and a negative correlation between the magnitude of connectivity and body size misjudgment [67]. Vocks and colleagues observed reduced activation in the inferior parietal lobule when patients viewed their own body, but increased activation of the amygdala when viewing pictures of another woman’s thin body [63]. The authors hypothesize correlations between decreased attentional processing towards the patient’s own body and an increased emotional response when viewing other bodies with an inherent bias towards social comparison. 

When asked to *compare their own body to slim, idealized female bodies*, patients with AN display reduced activation in the rostral anterior cingulate cortex, but increased activation in the insula and premotor cortex [64]. Thus, body image perception in patients with AN may be related to alterations in regions associated with interoceptive awareness. Furthermore, participants who have recovered from AN show increased activation in the rostral anterior cingulate cortex during a comparison of their own body with an underweight body [68]. This change may be viewed as a recovery of top-down control of the emotional impact of body comparisons with others. Fladung and colleagues [66] employed pictures of a female body corresponding to different weight categories; when patients with AN were asked to imagine having the same weight as featured in the picture, they showed increased activation in the bilateral ventral striatum when imagining having an underweight body shape compared to healthy controls. 

When shown pictures of their *own body digitally modified to be oversized*, patients with AN show increased activation in the dorsolateral prefrontal cortex compared to healthy controls [69]. Furthermore, this activation was related to eating disorder psychopathology (i.e., shape concerns). These results suggest an increase in top-down control when patients are facing emotionally aversive stimuli, such as distorted images of their own body. When asked to rate modified pictures of their own body, patients with AN display increased activation in the insula and lateral prefrontal cortex during the evaluation of thin self-images, which may indicate a stronger emotional involvement, although this claim remains inconclusive since both regions are associated with a number of differing functions [70,71,72]. 

Taken together, studies examining the passive viewing of body shapes, body comparison and modified images of one’s own body detected dysfunctional activation in neural body image networks and regions associated with interoception, and top-down control, as well as increased activation in regions associated with emotional processing and reward. Furthermore, the existing studies indicate a correlation between reduced top-down control and body-comparison in patients with AN, whereas the viewing of modified images of their own body is characterized by increased top-down control. These results highlight the importance of disturbed body image processing in patients with AN, indicating that underweight body images possess a rewarding effect and an increased emotional relevance for patients with AN.

## 5. Emotional Processing

Dysfunctional emotional processing is prevalent in patients with AN and is related to both the onset and prevalence of the disorder [73,74]. Dietary restrictions and binging/purging may facilitate the avoidance or reduction of negative emotions [75,76,77]. Accordingly, a broad range of emotion regulation deficits have been observed in patients with AN [78,79] and have been shown to correlate with AN psychopathology [80,81]. For example, behavioral investigations suggest an increase in disorder-related thoughts during an induced negative mood [82]. However, vast differences in emotion regulation exist across different types of eating disorders. Patients with binge eating/purging compared to restrictive eating behaviour experience greater difficulties in emotion regulation in the domains of goal-directed behaviour, impulse control and have limited access to regulatory strategies. In fact, the emotion regulation profile of patients with AN-binge eating/purging type (AN-BP) is similar to patients diagnosed with bulimia nervosa [83]. In contrast, patients with restrictive AN (AN-R) experience greater difficulties in recognizing and expressing of emotions than patients with AN-BP [84]. 

When assessing neural emotional processing in response to *affective facial expressions*, Fonville and colleagues [85] observed increased activation of the fusiform gyrus in patients with AN during exposure to happy facial expressions. This observation may indicate the increased saliency of facial expressions in patients with AN. However, Cowdrey and colleagues [86] failed to identify differences during the neural processing of happy and fearful faces in participants who had recovered from AN, leading the authors to conclude that deficits in the processing of emotional faces in patients with AN might be state-dependent and improve with recovery. In contrast, Rangaprakash and colleagues [87] reported reduced connectivity between the amygdala and prefrontal cortex when patients who had recovered from AN viewed fearful facial expressions. These findings suggest that a decrease in top-down control during the processing of emotional facial expressions represents a trait marker of AN, indicating that aberrations in emotion regulation persist at neurobiological and behavioural levels [88] in weight-recovered patients with AN. Leppanen and colleagues [89] investigated neural responses to faces of infants displaying positive and negative emotions, and found that patients with AN exhibit increased prefrontal downregulation of limbic regions when viewing of positive emotions, but increased activation of the posterior insula when viewing of negative emotions. The latter finding suggests an increased saliency of negative emotions in patients AN, due to the frequently observed association of insula activation and emotion processing [90]. Taken together, studies employing facial stimuli commonly observe increased neural saliency processing and suggest the presence of different activation profiles, depending on the emotional valence of facial stimuli, where negative emotional stimuli tend to be associated with decreased top-down control. 

Neural processing during *social interactions* also appears to be impaired in patients with AN. During positive social interactions in an economic exchange game, patients with AN and participants who had recovered from AN displayed diminished neural responses in the precuneus and right angular gyrus. However, only patients with current AN showed reduced activation in the fusiform gyrus during negative interactions [91]. The authors discuss a potential role of aberrant neural responses during positive social interactions as a predisposing trait for the development of AN, whereas changes in the neural processing of negative interactions may be important for weight recovery following AN. Via and colleagues used a social judgement task where participants received feedback on whether other participants would like to meet them [92]. Patients exhibited reduced activation of the dorsal prefrontal cortex when receiving positive feedback, but hyperactivation of visual regions and, surprisingly, a positive correlation between reward-related brain regions and clinical severity scores when receiving negative feedback. These results suggest the presence of dysfunctional self-evaluative processes and reduced perceptions of social rewards, and finally, they highlight the importance of brain reward networks for pathological behaviors in patients with AN. Consistent with the frequently observed disturbances in interpersonal relationships in patients with AN [93], Maier and colleagues [94] found that both patients with AN and participants who had recovered from AN display decreased activation in the superior parietal cortex and a reduced responsivity in the dorsolateral prefrontal cortex when viewing pictures displaying intimate situations. Similarly, Miyake and colleagues [95] observed a negative relationship between alexithymia, or the inability to articulate and interpret emotional experiences, and activation of the amygdala and posterior and anterior cingulate cortices during the processing of negative interpersonal words. Furthermore, reduced processing in the medial prefrontal cortex during a theory of mind task negatively correlates with treatment outcome [96]. This finding further corroborates the observed relation between social functioning and therapy outcomes in patients with AN [97] and confirms the importance and potential effectiveness of social skills training and family-based interventions for patients with AN [98,99]. 

Altered neural processing during the *active regulation of emotions* has also been observed in patients with AN. According to Seidel and colleagues, when patients with AN are asked to “distance” themselves from aversive pictures, they display the same neural activation pattern as healthy controls, but increased activation of the amygdala and dorsolateral prefrontal cortex is detected when they passively view aversive pictures [100]. However, in a subsequent study, Seidel and colleagues [101] reported a positive correlation between activation of the ventral striatum during distancing from positive pictures and body-related rumination, but this activation pattern negatively correlates with negative affect and treatment outcomes in patients with AN. Although the neural regulation of emotions is partially preserved in patients with AN, the authors emphasized the need to focus on adaptive emotion regulation strategies during the treatment of AN. 

Taken together, studies investigating neural activation related to the processing of emotions in patients with AN reported contradictory results, but suggest increased salience processing and a concurrent dysfunctional top-down control during emotional processing. However, heterogeneity in studies investigating emotion regulation in patients with AN might explain the fact that more than 55% of patients with AN exhibit at least one comorbid disorder [102], with approximately a 73% lifetime prevalence of depressive disorders among patients with AN [103]. Comorbid depression is associated with increased emotion regulation difficulties [104] and researchers have not clearly determined how these comorbidities, particularly mood disorders, interact with the neural processing of emotional stimuli in patients with AN. In previous functional imaging studies, little emphasis was placed on the contributions of comorbid disorders, which might play an essential role when examining disorder-specific stimuli, as these disorders might moderate the patient’s emotion regulation capacities.

## 6. Interoceptive Processing

Interoception refers to the perception and integration of visceral and homeostatic signals representing internal physiological body states [105]. Altered interoceptive awareness is viewed as a vulnerability factor for the development of AN, where overactive cognitive control enables the development of maladaptive food habits that do not subserve the homeostatic weight balance [13]. Decreased interoceptive awareness is associated with alexithymia, which is the to describe and identify one’s own feelings [106]. Alexithymia is related to impaired emotional regulation and is as risk factor for the development and maintenance of AN [107]. Several neuropsychological models of alexithymia have been proposed. According to an early hypothesis proposed by MacLean [108], an altered communication between limbic and neocortical brain areas exists, leading to impairments in identifying and describing one’s own emotions and feelings. As shown in recent studies, individuals with alexithymia display decreased activation of limbic and paralimbic brain areas in response to affective stimuli or increased activity in somatosensory/sensorimotor areas. The former indicates a low emotional arousal to external emotional stimuli, while the latter suggests a hypersensitivity and overreliance on physical stimulation [109].

Employing an interoceptive attention task where participants must focus on *internal bodily sensations* such as heartbeat or stomach distension, Kerr and colleagues [110] observed altered activity in the insula in weight-restored patients with AN. Furthermore, insula activity during stomach interoception negatively correlates with eating disorder psychopathology and anxiety. The authors postulate a visceral hypersensitivity entailing increased perception of gastrointestinal discomfort during food consumption, particularly during the weight restoration process. In a subsequent study, Kerr and colleagues [23] investigated the effect of interoceptive sensation on the neural processing of food pictures in weight-restored patients with AN. During the neural processing of food pictures, stomach sensation ratings recorded before scanning positively correlated with activity in the insula, anterior cingulate cortex and amygdala, but negatively correlated with activity in the ventral pallidum and ventral tegmental area. The authors concluded that gastric sensations may interfere with food reward processing and may be related to an aversive response to food pictures in patients with AN.

Two studies have investigated neural processing during unpleasant bodily sensations in patients with AN. Strigo and colleagues [111] investigated the neural processing of *pain* in patients recovered from AN and observed increased activity in the insula and dorsolateral prefrontal cortex (DLPFC) during the anticipation of pain, whereas the experience of pain was associated with decreased insula but increased DLPFC activity. Increased insular activity during the anticipation of pain was also related to high levels of alexithymia. The authors concluded that the results suggest an abnormal integration of interoceptive signals, and the increase in DLPFC activity is an attempt to control the increased distress caused by the subsequent pain stimulation.

Similarly, Berner and colleagues assessed neural processing in women who had remitted from AN during an *inspiratory breathing load paradigm*, where participants’ breathing was intermittently restricted, causing mild discomfort [112]. When anticipating breathing restriction, patients displayed reduced insular activation, whereas they showed stronger activation of the striatum, cingulate cortex and prefrontal cortex during the actual breathing restriction. These results may reflect difficulties in predicting and adapting to changes in interoceptive states in patients with AN, and highlight eating restriction as a method for preventing unpredictable and/or unpleasant internal changes.

Bischoff-Grethe and colleagues investigated neural processing during a *pleasant affective touch* task (i.e., gentle strokes with a soft brush administered to the forearm or palm), where participants who had recovered from AN displayed an increased response in the right ventral mid-insula, but a decreased response in the same region during the anticipation of a pleasant touch [113]. These results indicate an impaired ability of patients with AN to predict and interpret physiological stimuli. Furthermore, since reduced activity in the insula during anticipation is related to increased harm avoidance and higher body dissatisfaction, aberrant interoceptive processing might contribute to an altered subjective body experience and avoidance behavior [113]. 

In summary, the results from studies investigating neural interoceptive processing have identified a pronounced relation between neural hypersensitivity to bodily sensations and concurrent avoidance behavior, which is partially mediated by dysfunctional prefrontal processing. Importantly, since all of the aforementioned studies recruited women who had recovered from AN, dysfunctional interoceptive processing might be a potential trait of AN.

## 7. Summary and Conclusions

This narrative review aimed to provide a general overview of the neuroimaging literature investigating symptom-specific neural processing in patients with AN. In recent years, a number of studies have investigated the neural profiles of different symptom-categories in patients with AN using fMRI. While the obtained results sometimes differ and certain disorder-related stimuli have not yet been investigated, the findings provide a better understanding of the underlying neurobiological correlates of AN. Based on the reviewed literature, we provide the following summary of the different symptom categories.

Neural processing of visual *food* cues in patients with AN is characterized by increased top-down control, enabling restrictive eating habits and a concurrent reduction in hedonic and somatosensory reactivity. On the other hand, studies using real tastants typically observe increased activation of brain areas related to saliency and reward processing, as well as emotional arousal, namely, a fear of weight gain and drive for thinness. Thus, although an increase in neural cognitive control in response to food cues allows patients to avoid food and consistently reduce their cravings for food, the actual consumption of food is associated with a number of disorder-specific neural reactions that collectively increase the saliency of food.

In patients with AN, *hunger* is associated with a decrease in neural motivational processing in response to food cues, and the observed activation patterns suggest an increase in emotional sensitivity related to a fear of weight gain. This finding is also consistent with the results from animal models suggesting that conditioned fear cues inhibit food consumption by food-deprived rats; therefore, signals from the amygdala may potentially override the homeostatic signaling of hunger in the hypothalamus and inhibit eating [114]. Therefore, the frequently observed strong effect of top-down control regions on food reward processing [115] has also been observed on homeostatic signals driving food consumption. 

When using stimuli related to *body image*, studies identified a number of aberrations in brain regions related to emotion and reward processing, as well as interoceptive processing and top-down control. Body images gain increased emotional relevance, and exposure to thin body figures can become rewarding. These results are consistent with the “reward contamination theory” proposed by Keating [49], which hypothesizes that the observed reduction in neural food reward processing is caused by a pronounced fear of weight gain, but illness-related stimuli and behaviors, such as emaciated body shapes or food restriction, become rewarding and activate reward-related brain regions in patients with AN [50]. Furthermore, the observed activation of brain regions related to interoception in patients with AN who are confronted with body images supports the hypothesis that dysfunctional interoceptive awareness is linked and contributes to body image concerns [116].

The processing of *emotions* in patients with AN is characterized by increased saliency processing and dysfunctional top-down control. These observations extend to visual depictions of emotional facial expressions, social interactions and emotion regulation but are more pronounced for negative emotional stimuli. Neural activation in corticolimbic regions during the processing of social stimuli and emotion regulation are related to treatment outcomes. However, a number of open questions related to emotional processing in patients with AN remain, such as the effect of the satiety state, since no neuroimaging study has yet investigated the specific interaction between the hunger state and neural emotional processing in patients with AN. An investigation of this interaction would be very interesting because dietary restriction represents an important method avoiding or reducing negative emotions in patients with AN [77].

The neural processing of *interoception* is related to a dysfunctional integration of interoceptive signals in the insula and an increased general neural sensitivity to interoceptive and somatosensory stimuli, which facilitates restrictive and avoidant behaviors in patients with AN. These results are consistent with previous studies showing that anxiety associated with food intake in patients with AN is related to intensified interoceptive sensations [117]. 

Taken together, studies using symptom provocation paradigms to assess disorder-specific neurobiological alterations in patients with AN observed a number of alterations in different brain networks. Some of these activation patterns are related to the acute phase of AN and some have been identified as state-independent risk factors. Specifically, an increase in top-down control observed in response to *visual food cues* appears to enable restrictive eating; a concurrent increase in the activation of reward- and emotion related areas during *gustatory stimulation* triggers disorder-specific thought patterns, such as a fear of weight gain. *Hunger* loses its ability to motivate food foraging but instead reinforces disorder-related behaviors. Dysfunctional neural processing of *body images* is a central feature of AN, where increased emotional and hedonic processing are prevalent, and neural activation during *emotional stimuli* indicates decreased top-down control but increased salience, particularly for negative stimuli. Finally, dysfunctional *interoceptive processing* is a trait of AN, where neural hypersensitivity to bodily sensations promotes avoidance behavior.

Studies investigating the neurobiological correlates of disorder-specific stimuli in patients with other psychiatric disorders might provide information about the pathophysiological mechanisms underlying shared symptomatology of psychiatric disorders. Given the high prevalence of comorbidities in patients with AN, studies investigating patients with anxiety disorders (e.g. obsessive-compulsive disorder or social phobia), affective disorders or substance abuse disorder can contribute to a better understanding of the psychopathology of AN. For example, patients with OCD show increased activation of fronto-striato-limbic regions and the amygdala upon exposure to symptom-provoking stimuli [118]. The authors concluded that amygdala hyperactivation in response to disorder-related stimuli reflects an exaggerated fear response. This finding is similar to the observed amygdala hyperactivation in patients with AN upon exposure to taste stimuli [15], food [22], and their own body image [65]. Similar findings of exaggerated limbic activation in response to disorder-specific stimuli have been observed in individuals with substance use disorder [119], panic disorder with agoraphobia [120] and social anxiety disorder [121]. These results suggest a potential shared functional neural basis of symptom-provoking stimuli across patients with different psychiatric disorders. However, researchers have not clearly determined whether these commonalities are a causal factor for the response to disorder-specific stimuli or reflect an overlap of symptomatology underlying the high prevalence of comorbidities. 

Conclusions drawn from neuroimaging data often remain ambiguous since using observed brain activation to infer conclusions about cognitive processes can be problematic. Specifically, reverse inference, or “reasoning backwards from brain activation to the engagement of a particular cognitive function” [122] is only valid when the selectivity of activation in the observed brain regions is high. Furthermore, a number of limitations are present in fMRI-studies and should be taken into account when drawing general conclusions. General limitations relate to small sample sizes and an almost exclusive focus on female patients with AN. Contradictory results across studies could in part be caused by differences in experimental paradigms and sample heterogeneity such as illness state (acute vs. recovered AN) and duration, pharmacological treatment and psychiatric comorbidities. A number of studies have included patients recovered from AN to avoid the confounding effects of starvation. However, this approach can produce misleading findings, since patients often continue to display core symptoms even after recovery [123,124] and the definition of recovery varies substantially in the literature [125]. Furthermore, AN is characterized by alexithymia and high prevalence rates of affective disorders which likely moderate neural response to disorder-related stimuli and tasks [126]. Finally, emotion regulation difficulties differ across subtypes of AN [84] and future neuroimaging studies should address the influence of both comorbid disorders as well as differences in emotion regulation strategies across the eating disorder spectrum. A combination of neurophysiological techniques could contribute to the understanding of the interactive effect of neurobiology and disorder eating behavior underlying the psychopathology of AN.

Our conclusions are preliminary, since no systematic literature search or formal assessment of methodological quality was performed. However, the aim of this review was to reconcile findings derived from differing neural stimuli and to provide a parsimonious account of the underlying neurobiological alterations associated with AN. Alterations in brain networks subserving various functions jointly contribute to AN-specific symptoms and behaviors. Studies investigating symptom-specific neural processing will provide a better understanding of the mechanisms underlying AN and important suggestions for targets for neurobiologically informed treatments. In fact, treatment options integrating neurobiological contributions have already been described [127,128]. 
